# Evaluation of a target region capture sequencing platform using monogenic diabetes as a study-model

**DOI:** 10.1186/1471-2156-15-13

**Published:** 2014-01-29

**Authors:** Rui Gao, Yanxia Liu, Anette Prior Gjesing, Mette Hollensted, Xianzi Wan, Shuwen He, Oluf Pedersen, Xin Yi, Jun Wang, Torben Hansen

**Affiliations:** 1 BGI-Shenzhen, Shenzhen, China; 2 The Novo Nordisk Foundation Center for Basic Metabolic Research, Faculty of Health Sciences, University of Copenhagen, Copenhagen, Denmark; 3 XiangYa Medical School, Central South University, Changsha, China; 4 Steno Diabetes Center, Gentofte, Denmark; 5 Faculty of Health Sciences, University of Southern Denmark, Odense, Denmark; 6 Department of Biology, University of Copenhagen, Copenhagen, Denmark

## Abstract

**Background:**

Monogenic diabetes is a genetic disease often caused by mutations in genes involved in beta-cell function. Correct sub-categorization of the disease is a prerequisite for appropriate treatment and genetic counseling. Target-region capture sequencing is a combination of genomic region enrichment and next generation sequencing which might be used as an efficient way to diagnose various genetic disorders. We aimed to develop a target-region capture sequencing platform to screen 117 selected candidate genes involved in metabolism for mutations and to evaluate its performance using monogenic diabetes as a study-model.

**Results:**

The performance of the assay was evaluated in 70 patients carrying known disease causing mutations previously identified in *HNF4A, GCK, HNF1A, HNF1B, INS,* or *KCNJ11*. Target regions with a less than 20-fold sequencing depth were either introns or UTRs. When only considering translated regions, the coverage was 100% with a 50-fold minimum depth. Among the 70 analyzed samples, 63 small size single nucleotide polymorphisms and indels as well as 7 large deletions and duplications were identified as being the pathogenic variants. The mutations identified by the present technique were identical with those previously identified through Sanger sequencing and Multiplex Ligation-dependent Probe Amplification.

**Conclusions:**

We hereby demonstrated that the established platform as an accurate and high-throughput gene testing method which might be useful in the clinical diagnosis of monogenic diabetes.

## Background

Since 1992, several genetic subtypes of monogenetic diabetes have been described in which gene mutations result in diabetes, primarily by causing beta-cell dysfunction [1]. Thus, patients previously categorized clinically as having maturity-onset diabetes of the young (MODY), permanent neonatal diabetes mellitus (NDM), or transient NDM, can now be classified by genetic sub grouping. Identification of the genetic subgroups is a prerequisite for appropriate treatment, genetic counseling, and prognostic information.

MODY, defined as dominantly inherited, young-onset (at least one family member with onset before 25 years of age), non-autoimmune and non-insulin dependent diabetes (no insulin treatment needed three or more years after diabetes diagnosis or measurable circulating C-peptide) [1,2], is the most common form of monogenetic diabetes. Molecular genetic findings have shown that MODY is very heterogeneous. To date, the MODY phenotype has been reported to be linked with mutations within 13 different genes: *HNF4A* (MODY1), *GCK* (MODY2), *HNF1A* (MODY3), *PDX1* (MODY4), *HNF1B* (MODY5), *NEUROD1* (MODY6), *KLF11* (MODY7), *CEL* (MODY8), *PAX4* (MODY9), *INS* (MODY10), *BLK* (MODY11) and very recently *ABCC8* (MODY12) and *KCNJ11* (MODY13) [3-5]. Approximately 70-80% of clinically diagnosed MODY patients carry mutations in one of these 13 genes, however, the remaining 20-30% are likely to carry disease causing mutations in yet unidentified genes [1,6-8]. Thus, the term MODY encompasses a group of clinically and genetically heterogeneous forms of beta cell dysfunction and in a few instances also insulin resistance which at a molecular level is defined by mutations in various genes [2,5].

NDM, a form of monogenic diabetes which is usually diagnosed within the first six months of life, has a rare incidence of one in 100,000 to 400,000 live births. Clinically, NDM can be divided into two subtypes: transient neonatal diabetes mellitus (TNDM) and permanent neonatal diabetes mellitus (PNDM). The most frequent cause of PNDM is due to mutations in *KCNJ11* or *ABCC8* which encode a subunit and a modulator of the ATP-sensitive potassium (K_ATP_) channels in the pancreatic beta-cell, respectively [9]. Patients with PNDM due to *KCNJ11* or *ABCC8* mutations may be treated with oral sulfonylurea instead of insulin [1].

The monogenic subset of the diabetic population is often mis-classified as type 1 diabetes (T1D) or type 2 diabetes (T2D) and thus is not offered optimal treatment regimens [10]. In Europe and USA, approximately 2-5% of T2D and 10% of familial T1D have previously been estimated to be misdiagnosed MODY cases [11,12], however, the exact incidence and prevalence of monogenic diabetes is not known. According to recent estimates, approximately 92 million Chinese are recognized diabetes patients, making China one of the world’s leading countries with regards to diabetes prevalence [13]. In the latest report, 27.7 million Chinese children and 334 million Chinese adults are estimated to be pre-diabetic or diabetic [14]. With this high diabetes prevalence and the known high prevalence of specific MODY forms in young onset diabetic patients in China [15,16], the prevalence of monogenic diabetes cases in China is likely to be accordingly high, and the importance of clinical differentiation should thus not be ignored. A high-throughput method with high accuracy to diagnose monogenic forms of diabetes is needed not only for a scientific purpose but also for medical application. Due to its high accuracy, Sanger sequencing is still the gold standard in terms of gene variant detection. However, when it comes to heterogeneous disorders, the method requires accurate clinical assessments and gene selection. If the “wrong gene” is tested, a (false) negative result is obtained and the testing procedure has to be repeated. In this case, the method is time consuming and expensive.

Here, we present the use of a gene testing panel (117 genes) based on a target region capture system coupled with next-generation sequencing (NGS) technology. The performance of the capture probe was tested by use of in-house YanHuang (YH) DNA [17]. The assay performance was validated by investigating six of the known monogenic diabetes genes (*HNF4A, GCK, HNF1A, HNF1B, INS* and *KCNJ11*) in 70 Danish patients carrying previously identified causative variants. All 70 causative variants were correctly identified. We therefore believe the established platform to be a high-throughput gene testing method which may be applied in clinical diagnosis of monogenic diabetes.

## Methods

### Samples

As a means to test the use of the platform, six YH samples were contributed from the Clinical Laboratory Center at Beijing Genomic Institute (BGI) [17], Shenzhen. For the validation of the method in relation to detection of known diabetes causing mutations, a total of 70 samples from unrelated Danish MODY and NDM probands collected at Steno Diabetes Center, Gentofte, Denmark, were examined. Prior to the participation in the study, informed consent was obtained from all subjects. The study was approved by the Ethical Committee of Copenhagen and was in accordance with the principles of the Declaration of Helsinki II.

### Target region array design

A customized oligonucletide probe was designed to capture whole genome regions (including exons, introns, and untranslated regions (UTRs)) of 117 genes for monogenic diabetes [3-5] and selected candidate genes referred to type 2 diabetes mellitus, metabolism of glucose and oral hypoglycemic agents, and obesity [2,18,19] (Additional file 1: Table S1). Hence, the total size of probe set was 4962226 bp. The probes (GenCap™ Enrichment, MyGenostics, USA) were approximately 60 bp in length and with a 5-10 bp overlap in restricted regions.

### DNA extraction, target region capture and next-generation sequencing

Methods for DNA extraction, target region capture, and NGS have previously been extensively described [20] and a brief experimental workflow is included in Additional file 1: Figure S1. In brief, genomic DNA was extracted from peripheral blood lymphocytes by standard procedures using QIAamp DNA Bloodmini kits (Qiagen, Germany). Next, 1 ug genomic DNA was fragmented by Covaris sonicator (Covaris S2, USA) to sizes of 150-300 bp and then purified. The blunt ends of the purified DNA fragments were then repaired, and A-tailing was added. The fragments were ligated overnight using standard Illumina paired-end (PE) adapter. The ligated products were then amplified through 4-cycle polymerase chain reactions (PCRs) using PE primers containing 8 bp index tags. The purified PCR products containing 3 ug DNA were hybridized to the GenCap™ probe (in solution) at 65°C for 22 hours using a PCR machine. The products were bound to a rotator for 1 hour at room temperature using Dynal Myone Streptavidin C1 magnetic beads (Invitrogen, USA), which had been activated beforehand, and the products were then washed with buffer according to the kit manual. The captured DNA libraries were amplified using 15-cycle PCRs, purified, and subsequently eluted in a 30 ul volume and subjected to Agilent 2100 Bioanalyzer and quantitative PCR to estimate the magnitude of enrichment. The final captured DNA libraries were sequenced using the Illumina HiSeq2000 Analyzers as PE 90 bp reads (following the manufacturer’s standard cluster generation and sequencing protocols), providing an average coverage depth for each sample of at least 100-fold.

### Data filtering and analysis

Image analysis, error estimation, and base calling were performed using the Illumina pipeline (version 1.3.4) with default parameters. Indexed primers were used to identify the different samples in the primary data. All unqualified reads (defined as reads either polluted by adapter, containing more than 10% nucleotides out of read length, having an average quality of less than 10, or having 50% bases with a quality value less than 5) were removed using a local dynamic programming algorithm. The remaining reads were aligned to the reference human genome (UCSC hg19) using Burrows-Wheeler Alignment Tool (BWA-0.5.9). Next, SNPs and indels were identified using SOAPsnp software 2.0 and SAMtools v1.4 while using the recommended parameters [21,22].

### FNFP assessment

The accuracy and precision of the targeted region capture sequencing were assessed by comparing SNPs identified in one of the 6 YH samples with those reported in the online YH database (http://yh.genomics.org.cn/). The remaining 5 YH samples were used as inter-control samples.

According to the YH database reference, an in-house pipeline was used to estimate the true positive (TP) ratio, true negative (TN) ratio, false positive (FP) ratio, and false negative (FN) ratio of all SNPs [23]. The accuracy and precision was calculated by (TP + TN)/(TP + FN + TN + FP) and TP/(TP + FN), respectively. In this calculation, only high quality SNPs fulfilling the following criteria were included: 1) SOAPsnp score ≥ 20; 2) Depth ≥ 20; 3) Percentage of reads supporting variation ≥ 28% [24].

### Functional annotation of genetic variants

The variants were functionally annotated using an in-house pipeline as well as the reported frequencies available from public databases (dbSNP 135, HapMap database, 1000 genome variants database, and a local control database) and categorized into either missense, nonsense, splice-site, insertion, deletion, synonymous or noncoding mutations. For all variants, the results were filtered using a quality value of single base sequencing ≥ 20. The variants were filtered to potential mutation candidates through: 1). Functional variants (insertion/deletion: in CDS and splicing region, SNP: nonsense, splice site and missense) and 2). Variants with an allele frequency below 0.01 in either of the public databases mentioned above. To validate the pre-screened samples, variants were initially filtered by six known disease-causing genes (*HNF4A, GCK, HNF1A, HNF1B, INS* and *KCNJ11*).

The identification of known pathogenic variants was based on mutations previously reported to cause MODY in the literature, in Locus Specific Mutation Databases (LOVD, http://grenada.lumc.nl/LSDB_list/lsdbs) or previously identified to segregate with diabetes in Danish MODY families. Novel variants considered to be pathogenic were either: 1) stop/frameshift variants; 2) missense mutations positioned in the amino acid conservative region across species; 3) splice-site variations fulfilling the GT-AT rules; or 4) predicted to be possibly damaging or disease-causing by more than two of the bioinformatic programs (SIFT, Sorting Intolerant From Tolerant, http://sift.bii.a-star.edu.sg/; PolyPhen-2, http://genetics.bwh.harvard.edu/pph2/; Mutation Taster, http://www.mutationtaster.org/; BDGP, Berkeley Drosophila Genome Project, http://www.fruitfly.org/seq_tools/splice.html) [25].

### Large deletions/duplications analysis

The depths of each region of a gene in different samples within the same sequencing lane are significantly correlated (r > 0.7), and the depth of each capture region was therefore used to calculate a z-score according to the following equation:

$$z=\frac{\bar{X}-\mu_{0}}{\sigma_{0}/\sqrt{n}}=\frac{{\text{mean}}_{\text{Depth}\;\text{rate}‒\text{region}}-{\text{mean}}_{\text{Depth}\;\text{rate}‒\text{total}}\;}{S\text{tandard}\;D\text{eviation}}$$

Formula.1 z-score is calculated for large deletions/duplications analysis. Mean Depth rate-region= $\frac{\text{Mean}\;{\text{Depth}}_{\text{each}\;\text{region}}}{\text{Mean}\;{\text{Depth}}_{\text{each}\;\text{sample}}}$; Mean Depth rate-total= $\frac{\sum \text{Depth}\;{\text{rate}}_{\text{other}\;\text{region}}}{N}$, other region means other regions of the samples operated in the same run.

The large deletions and duplications were identified using a predefined cut-off point (±3) of derived z-score of each captured gene region. We used the cut-off value of 3 for absolute z-score, as it represents the 99.9^th^ percentile of the normal samples set for one tailed region. Any region with a z-score above 3 was defined as either a deletion (<-3) or a duplication (> 3).

## Results

### General performance of the overall sequencing

**Figure 1:**
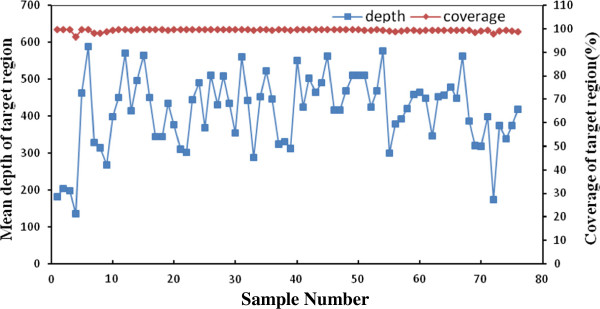
**Depth and coverage of all pre-screened samples.** For each screened sample (X-axis), the sequencing depth and the coverage of at least 1-fold depth are depicted as square (■) and diamond (♦), respectively. The Y-axis are Mean Depth (left) and coverage (right) respectively.

In total, we collected 76 samples to assess the method of target region capture combined with high-throughput sequencing, the brief analysis workflow can be found in supplementary information (Additional file 1: Figure S1) and the average performance of the method is provided in Table 1. For each sample, we obtained an average of 4409.95 Mb raw data of which 2046.69 Mb was mapped to the target region after alignment using BWA. The specificity of the probe was 53.72%, the overall coverage of the target region was 99.37% and 96.95% for a minimum depth of 1X and 20X, respectively, and the average sequencing depth was 412-fold (Table 1). For the 200 bp flanking target regions, the coverage was 94.45%, and the average depth was 240-fold (Table 1). The mean depth and the coverage of the target region for each screened sample are provided in Figure 1.

**Table 1:** Statistics of the performance

**Target region capture statistics**	**Value**
Target region (bp):	4962226
Raw data yield (Mb)	4409.95
Data mapped to target region (Mb):	2046.69
Reads mapped to genome:	48535673.13
Reads unique mapped to genome:	46489667.46
Reads mapped to target region:	25879158.93
Mean depth of target region:	412.45
Coverage of target region > =1X (%):	99.37
Coverage of target region > =20X (%):	96.95
Average read length (bp):	89.11
Capture specificity (%):	53.72
Mean depth of flanking region:	240.00
Coverage of 200 bp flanking region (%):	94.45

The target region capture performance is presented in average value of all 76 the sequenced samples.

Using SOAPsnp and SAMtools for variant calling [21,22], we obtained a total of 15875 variants in the 117 genes on average pr. sample, including 13808 SNPs and 2067 indels. All variants were annotated as either heterozygous (het) or homozygous (hom) using the bioinformatics software (hom-0.0005, het-0.0010) [21]. Overall, 96.83% and 1.12% of SNPs were located in the introns and exons, respectively (Table 2), and the percentage of heterozygotic and homozygotic SNPs was 70.94% and 29.06%, respectively (Table 2). In each screened sample, an average of 70 missense, 18 splice site, 1 nonsense, and 66 synonymous mutations were identified.

**Table 2:** Statistics of the SNP count

**SNP classification**	***n***	**Ratio (%)**	**SNP classification**	***n***	**Ratio (%)**
Missense	70.01	0.51	Hom	4012.28	29.06
Splice-site	17.97	0.13	Het	9795.97	70.94
Nonsense	0.79	0.01			
Synonymous	66.28	0.48			
5′UTRs	23.45	0.17			
3′UTRs	187.75	1.36			
Intron	13370.00	96.83			
Intergenic	72.00	0.52			
Read-through	0.00	0.00			
Total number of SNPs			13808.25		

The average number of single nucleotide polymorphisms (in different categories) identified by sequencing 117 genes in 76 samples.

With regards to the indels, 97.28% and 0.2% were located in introns and exons, respectively, and the percentage of heterozygotic and homozygotic indels was 93.27% and 6.73%, respectively (Table 3). Furthermore, on average, each sample had a total of 1226 deletions and 841 insertions in the all gene region, 3 deletions and 1 insertion positioned within a coding region, and 1 indel positioned in a splice site region.

**Table 3:** Statistics of the indel count

**Indel classification**	***n***	**Ratio (%)**	**Indel classification**	***n***	**Ratio (%)**	**Indel classification**	***n***	**Ratio (%)**
Del-coding	2.93	0.14	Het InDels	1927.41	93.27	Total deletion	1225.63	59.31
Ins-coding	1.26	0.06	Hom InDels	139.14	6.73	Total insertion	840.92	40.69
Splice-site	0.80	0.04						
Intron	2030.18	97.28						
5′UTRs	3.00	0.14						
3′UTRs	47.00	2.25						
Promo	1.68	0.08						
Intergenic	0.00	0.00						
Total number of indels				2066.55				

The average number of small deletions and insertions (< 10 bp) identified by sequencing 117 genes in 76 samples.

### Accuracy and precision of target region captured sequencing

To assess the accuracy of the method, we used the YH DNA as a control sample, as genome information of this sample was readily available [17]. In this sample, the relative TP, TN, FN and FP ratio for all SNPs were 90.96%, 99.99%, 9.04%, and 0.006%, respectively. The precision of sequencing was 95.43%, and the accuracy was 99.98% (Table 4).

**Table 4:** The accuracy and precision assessment

**Filtered conditions**	**SOAPsnp score ≥ 20; Depth ≥ 20;**
	**Percentage of reads supporting ≥ 28%**
Target covered:	4944470 bp
Total number of SNPs:	9572
Total number of target SNPs:	6127
YH target in dbSNP:	6463
True positive (TP ratio):	5847 (90.96%)
True negative (TN ratio):	4937762 (99.99%)
False negative (FN ratio):	581 (9.04%)
False positive (FP ratio):	280 (0.006%)
Accuracy (%)((TP + TN)/(TP + FN + TN + FP)):	99.98
Precision (%)(TP/(TP + FN)):	95.43

The assessment is calculated based on qualified SNPs filtered by SOAPsnp score, depth, and supporting reads ratio.

### Identified variants in monogenic diabetes samples

To validate whether the method can accurately detect causative variants in patients with known disease causing mutations in *HNF4A, GCK, HNF1A, HNF1B, INS,* or *KCNJ11*, we applied the method to 70 samples from Danish patients.

**Figure 2:**
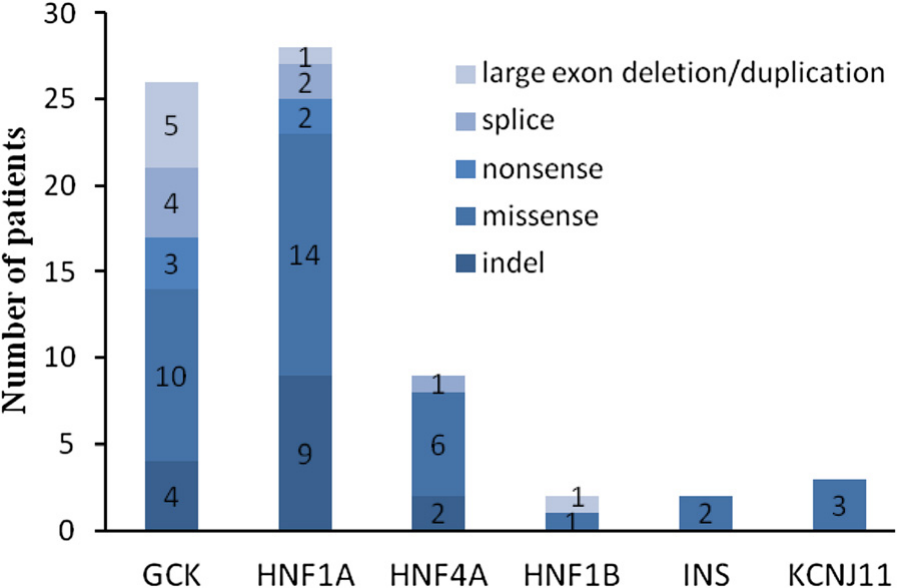
**The distribution of pathogenic (suspected pathogenic) variants of disease-causing genes among 70 pre-screened samples.** The mutation type (large exon deletion/duplication, splice, nonsense, missense, small indel) are present by gradually changing color in blue.

In regions with a more than 20-fold minimum depth, the coverage was 97.87%. Regions with a less than 20-fold depth were either introns or UTRs. Moreover, when only considering translated regions, the coverage was 100% with a 50-fold minimum depth. Thus, by selecting only genes of relevance for the individuals phenotype combined with data filtering, the number of potential pathogenic variants (indel, missense, nonsense, and splice-site) [5,23] in each sample could be narrowed down to 3-10 variants. These variants were arbitrarily defined to be either pathogenic, suspected pathogenic, polymorphism or of unknown significance. Mutations previously reported in MODY patients, or leading to loss of function due to the introduction of a stop codon or a frameshift, were termed pathogenic (n = 51). For novel mutations, functional prediction was performed with the use of bioinformatics tools. If novel mutations were located in important functional regions and/or conservative regions, they were defined as suspected pathogenic (n = 12). Polymorphisms, i.e. variants detected in both diabetes patients and controls, were predicted to be benign, and the remaining variants were defined as being of unknown significance. Hence, in 70 positive samples, we identified 63 pathogenic variants, including 15 indels, 36 missense, 5 nonsense, and 7 splice-site variants (Figure 2 and Additional file 1: Table S2). In the remaining 7 samples, we identified large deletions or duplications in exons (Additional file 1: Table S2, Additional file 1: Figure S3). Among the 70 samples, the percentage of *GCK*-mutated samples was 37%, *HNF1A* was 40%, *HNF4A* was 13%, *HNF1B* was 3%, *INS* was 3%, and *KCNJ11* was 4% (Additional file 1: Table S2). In addition, a series of polymorphisms and variants of unknown significance located within these genes was also identified.

### The clinical characteristics of Danish patients of monogenic diabetes mellitus

The clinical characteristics were collected at Steno Diabetes Center, Denmark and included the pathogenic gene, sex, age at examination and diagnosis, height, weight, BMI, HbA1c and treatment of every patient (Table 5). All 70 patients were diagnosed with diabetes mellitus at a young age, except one patient who was diagnosed with hyperinsulinemic hypoglycemia. Furthermore, all patients had a normal BMI and slightly elevated levels of HbA1c, and these clinical characteristics are all corresponding to the known features of monogenic diabetes. With the use of target region capture NGS, 100% of pathogenic variants previously identified by Sanger sequencing and MLPA were detected in a study sample of 70 Danish patients with known monogenic forms of disease. Therefore, the targeted NGS approach was demonstrated to be an accurate and reliable method to detect variants of monogenic diabetes or hyperinsulinemic hypoglycemia.

**Table 5:** Phenotype information

***Gene***	**n**	**Sex (m/f)**	**Age at examination (years) [NA]**	**Age at diagnosis (years) [NA]**	**Height (cm) [NA]**	**Weight (kg) [NA]**	**BMI (kg/m**^**2**^**) [NA]**	**HbA1c (%)**	**Treatment (ins/ins + OHA/OHA/diet) [NA]**
*GCK*	26	10/16	23.84 (1.4-73) [1]	16.26 (1.4-35) [3]	155.55 (80-176) [5]	55.53 (12-95.5) [4]	21.63 (13.46-37.59) [4]	6.56 (5.9-8.1) [3]	1/1/5/17 [2]
*HNF1A*	28	14/14	35.14 (8-60) [0]	23.24 (8-49) [3]	171.45 (155-188.5) [8]	71.48 (50.8-106.6) [8]	24.26 (18.82-37.77) [8]	7.42 (5.5-9.4) [8]	7/1/10/5 [5]
*HNF1B*	2	0/2	29.5 (26-33) [0]	29 (26-32) [0]	167 (162-172) [0]	58.25 (55.4-61.1) [0]	21.00 (18.73-23.28) [0]	6.05 (5.6-6.5) [0]	1/0/1/0 [0]
*HNF4A*	9	1/8	22.33 (4-35) [0]	15.67 (4-34) [0]	157.19 (96.5-183) [1]	57.34 (15.4-81.3) [1]	22.17 (16.54-25.59) [1]	7.09 (5.5-8.4) [1]	3/1/0/5 [0]
*INS*	2	2/0	39.5 (35-44) [0]	10.6 (0.2-21) [0]	171.5 (169-174) [0]	72.2 (71-73.4) [0]	24.55 (24.24-24.86) [0]	6.8 (5.9-7.7) [0]	1/0/1/0 [0]
*KCNJ11*	3	2/1	6.83 (3.5-13) [0]	0.33 (0.2-0.5) [0]	131.5 (108-155) [1]	29.9 (20.8-39) [1]	17.03 (16.23-17.83) [1]	7.37 (6.4-8.1) [0]	3/0/0/0 [0]
Total	70	29/41	28.11 (1.4-73) [1]	18.38 (0.2-49) [6]	161.69 (80-188.5) [15]	61.26 (12-106.6) [14]	22.58 (13.46-37.77) [15]	6.96 (5.5-9.4) [12]	16/3/17/27 [7]

The clinical characteristics of 70 Danish patients with known mutations in *GCK*, *HNF1A*, *HNF1B*, *HN4A*, *INS* or *KCNJ11*.

## Discussion

In the present study, target region enrichment combined with NGS technology proved to be a useful tool for the identification of genetic variants involved in monogenic diabetes. The hybridizing procedure to enrich target region is easy to use, and the duration of the total process is approximately 30 hours which is considerably less than performing directional sequencing of each gene. Combined with the applied sample pooling strategy, the target region capture procedure proved to be very effective. With the use of this method, 117 genes potentially implicated in metabolic disorders could be analyzed in one run. Each sample generated an average of 2005.13 Mb data mapped to the target region. Though the capture specificity of the applied chip is relatively low (53.72% compared to ~70% on other commercial chips, e.g. Nimblegen EZ Choice), the compatible performance in capture coverage (reducing the FN rate) as well as the lower cost (20% more sequencing cost is also considered) makes the present capture chip a good choice for target-region capture sequencing. For the entire testing panel, the coverage of the 117 genes was 99.38%, and the coverage was only slightly diminished (96.95%) at 20-fold minimum depth.

To assess the accuracy of the method, in-house YH DNA was initially tested. We found that after filtering of the low quality variants, the accuracy was 95%, and both the FP and FN ratio reached the lowest value. Nevertheless, comparison with the reference data revealed that the FN ratio was still quite high (9.04%). The relatively high FN ratio may be due to errors in the YH reference sequence; such false positives in the reference sequence will cause an inflated FN rate in the examined sequence. This is supported by the fact that an FN ratio of 5-10% has also been observed in other previous studies using the YH reference sequence [23].

By applying this method, a total of 70 pre-screened samples were tested, and in 100% of the samples, we were able to detect known mutations. With the use of this method, small variants (both SNPs and indels) as well as larger deletions and duplications could be identified in one analysis. In order to apply the method in clinical settings, several improvements could be implemented. Firstly, the gene panel could be re-designed to include only translated regions of phenotype specific genes (known causative genes, candidate genes for MODYX, other disease functional related genes), plus regulatory regions with reported causative mutations, for example the P2 promoter of *HNF4A*[26]. Secondly, any low coverage regions should be supplemented by Sanger sequencing methods in order to improve the mutation detection rate. Thirdly, our assay at its current form has a turnaround time of seven weeks, which is relatively long for routine clinical testing and reporting. Therefore, transferring the sequencing procedure from Hiseq to Miseq or Proton might shorten turnaround time while maintaining the high accuracy and throughput. Last but not least, clinical variation interpretation can be a great challenge. Thus, we suggest a “phenotype-specific candidate-genes panel” approach i.e. only genes of relevance to the individuals’ phenotype are analysed in order to reduce the number of variants identified to facilitate the interpretation of the pathogenicity of the variants [27]. A similar approach was recently published targeting the coding regions of 29 genes in which mutations have been reported to cause neonatal diabetes, MODY, maternally inherited diabetes and deafness (MIDD) or familial partial lipodystrophy (FPLD) [28]. However, as the present platform includes a much larger number of genes, includes non-coding region, and has the ability to identify larger structural variations, we believe that the present platform has a greater usability.

## Conclusions

We have established a straight-forward target region sequencing method for examining a large number of genes. The method was successfully evaluated using monogenic diabetes as a study-model. The turnaround time was seven weeks, and the coverage of the target gene coding region reached 100%. The method can be used to detect small size mutations and larger deletions and/or duplications in the same run. In terms of the 70 pre-screened samples, we correctly identified all the causative variants. We believe our method could be a useful tool in large-scale monogenic diabetes gene testing.

## Abbreviations

BGI: Beijing Genomic Institute; BMI: Body mass index; FN: False negative; FP: False positive; Het: Heterozygous; Hom: Homozygous; Ins: Insulin; LOVD: Locus specific mutation databases; MALDI-TOF-MS: Matrix-Assisted Laser Desorption/Ionization time-of-flight mass spectrometry; MLPA: Multiplex ligation-dependent probe amplification; MODY: Maturity-Onset Diabetes of the Young; NA: Not applicable; NDM: Neonatal diabetes mellitus; NGS: Next generation sequencing; PCR: Polymerase chain reaction; PE: Paired-end; PHHI: Persistent hyperinsulinemic hypoglycemia of infancy; PNDM: Permanent neonatal diabetes mellitus; OHA: Oral hypoglycemic agents; SNP: Single nucleotide polymorphism; T1D: Type 1 diabetes; T2D: Type2 diabetes; TN: True negative; TNDM: Transient neonatal diabetes mellitus; TP: True positive; UTR: Untranslated region; YH: YanHuang.

## Competing interests

The authors declare that there is no conflict of interest associated with this manuscript.

## Authors’ contributions

RG participated in the design of the study, performed the statistical analysis and drafted the manuscript. YXL performed all the bioinformatics analysis and part of the manuscript writing. XZW and SWH carried out the target capture experiments. MH and ARG carried out the pre-screening and clinical information analysis. TH, OP and XY conceived of the study and participated in its design and coordination and helped to draft the manuscript. All authors read and approved the final manuscript.

## Supplementary Material

Additional file 1: Table S1 The examined genes on designed capture panel. **Table S2.** Overview of the pathogenic variants of six monogenic diabetes mellitus’ casual genes. **Figure S1.** Workflow of experimental procedure before sequencing. **Figure S2.** Bioinformatics analysis pipeline. **Figure S3.** Detection of 7 large deletions or duplications in Danish patients with known monogenic diabetes. YH4 is shown as control. Click here for file
