# Supplementary material for: Evaluation of a target region capture sequencing platform using monogenic diabetes as a study-model
Source: BMC Genet. 2014 Jan 29;15:13. doi: 10.1186/1471-2156-15-13 (PMC3943834; doi:10.1186/1471-2156-15-13)
Supplement: Additional file 1: Table S1 — The examined genes on designed capture panel. Table S2. Overview of the pathogenic variants of six monogenic diabetes mellitus’ casual genes. Figure S1. Workflow of experimental procedure before sequencing. Figure S2. Bioinformatics analysis pipeline. Figure S3. Detection of 7 large deletions or duplications in Danish patients with known monogenic diabetes. YH4 is shown as control. [file 1471-2156-15-13-S1.docx]

**Table S1- The examined genes on designed capture panel**

| Number | Gene |
| --- | --- |
| 1 | ABCB1 |
| 2 | ABCB11 |
| 3 | ABCC2 |
| 4 | ABCC8 |
| 5 | ABCG2 |
| 6 | ADIPOQ |
| 7 | ADIPOR1 |
| 8 | ADIPOR2 |
| 9 | AGPAT2 |
| 10 | AIPL1 |
| 11 | ALMS1 |
| 12 | BCDIN3D |
| 13 | BDNF |
| 14 | BMP2 |
| 15 | BSCL2 |
| 16 | CAPN10 |
| 17 | CAV1 |
| 18 | CEL |
| 19 | CLK3 |
| 20 | CREB1 |
| 21 | CYP2C19 |
| 22 | CYP2C8 |
| 23 | CYP2C9 |
| 24 | CYP2D6 |
| 25 | CYP3A4 |
| 26 | EIF2AK3 |
| 27 | ETV5 |
| 28 | FOXO1 |
| 29 | FOXP3 |
| 30 | FTO |
| 31 | GATA6 |
| 32 | GCG |
| 33 | GCK |
| 34 | GCKR |
| 35 | GIPR |
| 36 | GLIS3 |
| 37 | GLUD1 |
| 38 | GNPDA2 |
| 39 | GZMB |
| 40 | GZMH |
| 41 | HADH |
| 42 | HFE |
| 43 | HNF1A |
| 44 | HNF1B |
| 45 | HNF4A |
| 46 | HYMAI |
| 47 | IGFL4 |
| 48 | INS |
| 49 | INSR |
| 50 | ITLN1 |
| 51 | ITLN2 |
| 52 | KCNJ11 |
| 53 | KCTD15 |
| 54 | LEP |
| 55 | LEPR |
| 56 | LM- |
| 57 | LRAT |
| 58 | LYPLAL1 |
| 59 | MC4R |
| 60 | MEF2A |
| 61 | MEF2D |
| 62 | MNX1 |
| 63 | MSRA |
| 64 | MTCH2 |
| 65 | MTNR1B |
| 66 | NDN |
| 67 | NEGR1 |
| 68 | NEUROD1 |
| 69 | NKX2-2 |
| 70 | NOS1AP |
| 71 | NRXN3 |
| 72 | O3FAR1 |
| 73 | OPA1 |
| 74 | PCK1 |
| 75 | PCK2 |
| 76 | PDX1 |
| 77 | PKLR |
| 78 | POMC |
| 79 | PPARA |
| 80 | PPARG |
| 81 | PPARGC1A |
| 82 | PPARGC1B |
| 83 | PRKAA1 |
| 84 | PRKAA2 |
| 85 | PRKAB1 |
| 86 | PRKAB2 |
| 87 | PRKAG1 |
| 88 | PRKAG2 |
| 89 | PRKAG3 |
| 90 | PTF1A |
| 91 | PTPN1 |
| 92 | PYY |
| 93 | RFX6 |
| 94 | SEC16B |
| 95 | SERPINB3 |
| 96 | SERPINB4 |
| 97 | SH2B1 |
| 98 | SIM1 |
| 99 | SLC19A2 |
| 100 | SLC22A1 |
| 101 | SLC22A2 |
| 102 | SLC22A3 |
| 103 | SLC22A8 |
| 104 | SLC29A4 |
| 105 | SLC47A1 |
| 106 | SLC47A2 |
| 107 | SLCO1B1 |
| 108 | SLCO1B3 |
| 109 | SLCO4C1 |
| 110 | SNRPN |
| 111 | SPATA7 |
| 112 | STK11 |
| 113 | TFAP2B |
| 114 | TMEM18 |
| 115 | WFS1 |
| 116 | ZACN |
| 117 | ZMPSTE24 |

**Table S2 –Overview of the pathogenic variants of six monogenic diabetes mellitus’ casual genes.**

| **Function** | **Sample** | **Gene** | **Chr** | **SNP rs** | **Region** | **DNA change** | **Animo acid change** | **Ref** | **Software prediction of variants** | | | | |
| --- | --- | --- | --- | --- | --- | --- | --- | --- | --- | --- | --- | --- | --- |
|  |  |  |  |  |  |  |  |  | SIFT | | PolyPhen-2 | | MutationTaster |
| Indel | M127-2 | GCK | 7 | - | Exon6 | c.608_609delTG | p.Val203fsdelTG |  |  |  | | disease causing | |
|  | M1169-1 | GCK | 7 | - | Exon8 | c.1005..1006delGT | p.Val335fsdelGT |  |  |  | |  | |
|  | M1026-1 | GCK | 7 | - | Exon9 | c.1210_1211delAT | p.Ile404fsdelAT |  |  |  | | disease causing | |
|  | M1116-1 | GCK | 7 | - | Exon10 | c.1340..1341insCCG | p.Arg447fsinsCCG | [1] |  |  | | disease causing | |
|  | M1093-1 | HNF1A | 12 | - | Exon3 | c.631_633delCAG | p.Gln211fsdelCAG |  |  |  | | disease causing | |
|  | M27-1 | HNF1A | 12 | - | Exon4 | c.873delA | p.Pro291fsdelA | [2] |  |  | | disease causing | |
|  | M1179-1 | HNF1A | 12 | - | Exon4 | c.863_864insC | p.Gly288fsinsC |  |  |  | | disease causing | |
|  | M1175-1 | HNF1A | 12 | - | Exon6 | c.1120delG | p.Gly374fsdelG | [3] |  |  | | disease causing | |
|  | M157-1 | HNF1A | 12 | - | Exon6 | c.1136_1137delCT | p.Pro379fsdelCT | [4] |  |  | | disease causing | |
|  | M2-1 | HNF1A | 12 | - | Exon6 | c.1137delT | p.Pro379fsdelT | [5] |  |  | | disease causing | |
|  | M12-14 | HNF1A | 12 | - | Exon9 | c.1676_1677insA | p.Ala559fsinsA | [6, 7] |  |  | | disease causing | |
|  | M115-1 | HNF1A | 12 | - | Exon9 | c.1766_1767insA | p.Thr589fsinsA | [3] |  |  | | disease causing | |
|  | M126-1 | HNF1A | 12 | - | Exon9 | c.1634_1637delCAGA | p.Ser545fsdel CAGA | [3] |  |  | | disease causing | |
|  | M21-1 | HNF4A | 20 | - | Exon2 | c.184delT | p.Phe62fsdelT | [8] |  |  | | disease causing | |
|  | M34-1 | HNF4A | 20 | - | Exon5 | c.489_490insG | p.Asp164fsinsG |  |  |  | | disease causing | |
| Missense | M67-1 | GCK | 7 | - | Exon4 | c.365T->C | p.Leu122Pro | [9] | damaging | probably damaging | | disease causing | |
|  | F302-3 | GCK | 7 | - | Exon5 | c.933G->A | p.Gly178Glu | [1] | damaging | probably damaging | | disease causing | |
|  | M1063-1 | GCK | 7 | - | Exon6 | c.617C->T | p.Thr206Met | [9][10] | damaging | probably damaging | | disease causing | |
|  | M1094-1 | GCK | 7 | - | Exon6 | c.660C->G | p.Cys220Trp | [11] | damaging | probably damaging | | disease causing | |
|  | M1048-1 | GCK | 7 | - | Exon7 | c.773G->A | p.Gly258Asp | [12] | damaging | probably damaging | | disease causing | |
|  | M1159-1 | GCK | 7 | - | Exon7 | c.860A->C | p.Gln287Pro | [13] | damaging | probably damaging | | disease causing | |
|  | M1051-1 | GCK | 7 | - | Exon8 | c.875T->A | p.Leu292His | [1] | damaging | probably damaging | | polymorphism | |
|  | M1225-1 | GCK | 7 | - | Exon9 | c.1067G->T | p.Gly356Val |  | damaging | probably damaging | | disease causing | |
|  | M1163-1 | GCK | 7 | - | Exon9 | c.1231T->C | p.Ser411Pro | [14] | damaging | probably damaging | | disease causing | |
|  | M62-1 | GCK | 7 | rs104894014 | Exon10 | c.1367C->T | p.Ala456Val | [15] | damaging | possibly damaging | | disease causing | |
|  | M69-1 | HNF1A | 12 | - | Exon1 | c.34C->T | p.Leu12Phe | [16] | damaging | probably damaging | | disease causing | |
|  | M52-1 | HNF1A | 12 | - | Exon1 | c.144G->A | P.Glu48Lys | [3, 17] | tolerated | benign | | polymorphism | |
|  | M6-1 | HNF1A | 12 | - | Exon2 | c.427C->T | p.His143Tyr | [18] | damaging | probably damaging | | disease causing | |
|  | M29-1 | HNF1A | 12 | - | Exon2 | c.476G->A | p.Arg159Gln | [19] | damaging | probably damaging | | disease causing | |
|  | M1021-1 | HNF1A | 12 | - | Exon3 | c.607C->T | p.Arg203Cys | [2] | damaging | probably damaging | | disease causing | |
|  | M125-1 | HNF1A | 12 | - | Exon3 | c.608G->A | p.Arg203His | [20][21] | damaging | probably damaging | | disease causing | |
|  | M25-1 | HNF1A | 12 | - | Exon3 | c.626G->C | p.Arg229Pro | [16] | damaging | probably damaging | | disease causing | |
|  | M51-8, M1151-1 | HNF1A | 12 | - | Exon4 | c.721T->G | p.Cys241Gly | [17] | damaging | probably damaging | | disease causing | |
|  | M118-1 | HNF1A | 12 | - | Exon4 | c.779C->T | p.Thr260Met | [18][21] | damaging | probably damaging | | disease causing | |
|  | M1066-1 | HNF1A | 12 | - | Exon4 | c.788G->A | p.Arg263His | [19] | damaging | probably damaging | | disease causing | |
|  | M14-1 | HNF1A | 12 | - | Exon4 | c.811C->T | p.Arg271Trp |  | damaging | probably damaging | | disease causing | |
|  | M1200-1 | HNF1A | 12 | - | Exon4 | c.812G->A | p.Arg271Gln | [16][22] | tolerated | probably damaging | | disease causing | |
|  | M3-S11 | HNF1A | 12 | rs137853236 | Exon7 | c.1340C->T | p.Pro447Leu | [7][23] | damaging | probably damaging | | disease causing | |
|  | M1168-1 | HNF4A | 20 | - | Exon2 | c.139G->A | p.Gly47Ser |  | damaging | probably damaging | | disease causing | |
|  | M1153-1 | HNF4A | 20 | - | Exon2 | c.187C->T | p.Arg63Trp | [24] | damaging | probably damaging | | disease causing | |
|  | M1189-1 | HNF4A | 20 | - | Exon3 | c.242T->G | p.Val81Gly |  | damaging | probably damaging | | disease causing | |
|  | M1014-1 | HNF4A | 20 | - | Exon4 | c.334C->T | p.Arg112Trp | [25] | damaging | probably damaging | | disease causing | |
|  | M131-1 | HNF4A | 20 | - | Exon8 | c.863G->A | p.Arg288Gln | [3] | tolerated | benign | | disease causing | |
|  | M1006-1 | HNF4A | 20 | - | Exon10 | c.1244C->T | p.Pro415Leu | [26] | damaging | probably damaging | | disease causing | |
|  | M1162-1 | HNF1B | 17 | - | Exon4 | c.883C->T | p.Arg295Cys | [27] | damaging | probably damaging | | disease causing | |
|  | M132-1 | INS | 11 | rs121908259 | Exon1 | c.17G>A | p.Arg6His | [28] | tolerated | benign | | polymorphism | |
|  | 100100030 | INS | 11 | - | Exon2 | c.284G->A | p.Cys95Tyr | [29] | damaging | probably damaging | | disease causing | |
|  | M1056-1 | KCNJ11 | 11 | rs80356625 | Exon1 | c.340C->T | p.Arg201Cys | [30] | damaging | probably damaging | | disease causing | |
|  | M1028-1 | KCNJ11 | 11 | rs80356624 | Exon1 | c.341G->A | p.Arg201His | [30] | damaging | probably damaging | | disease causing | |
|  | M1096-1 | KCNJ11 | 11 | - | Exon1 | c.145C->T | p.Arg163Cys | [31] | damaging | probably damaging | | disease causing | |
| Nonsense | M1103-1 | GCK | 7 | - | Exon8 | c.891C->A | p.Tyr297X | [32] |  |  | | disease causing | |
|  | M113-1 | GCK | 7 | - | Exon9 | c.1183G->T | p.Glu395X | [3] |  |  | | disease causing | |
|  | M1150-1 | GCK | 7 | - | Exon10 | c.1322C->A | p.Ser441X | [33] |  |  | | disease causing | |
|  | M1019-1 | HNF1A | 12 | - | Exon2 | c.370C->T | p.Gln124X | [3] |  |  | | disease causing | |
|  | M17-1 | HNF1A | 12 | - | Exon3 | c.700G->T | p.Glu234X | [34] |  |  | | disease causing | |
| Splice | M40-1 | GCK | 7 | - | Intron1 | c.45+1G->T |  | [9] |  |  | | disease causing | |
|  | M1042-1 | GCK | 7 | - | Intron4 | c.483+1G->A |  |  |  |  | | disease causing | |
|  | M90-1 | GCK | 7 | - | Intron8 | c.1019+1G->A |  | [9][35] |  |  | | disease causing | |
|  | M1216-1 | GCK | 7 | - | Intron3 | c.363+1G->A |  | [36] |  |  | | disease causing | |
|  | M1206-1 | HNF1A | 12 | - | Intron4 | c.956-2A->G |  | [37] |  |  | | disease causing | |
|  | M1027-1 | HNF1A | 12 | - | Intron8 | c.1623+1G->T |  | [37] |  |  | | disease causing | |
|  | M1128-1 | HNF4A | 20 | - | Intron5 | c.582+4A->G |  |  |  |  | | disease causing | |
| deletion | M8-1 | HNF1A | 12 | - | Exon2-10 |  |  | [38] |  |  | |  | |
|  | M1092-1 | HNF1B | 17 | - | Exon1-9 |  |  |  |  |  | |  | |
|  | M143-1 | GCK | 7 | - | Exon1-10 |  |  |  |  |  | |  | |
|  | M1173-1 | GCK | 7 | - | Exon1 |  |  |  |  |  | |  | |
| Duplication | M1035-1 | GCK | 7 | - | Exon2-3 |  |  |  |  |  | |  | |
|  | M159-4 | GCK | 7 | - | Exon2-3 |  |  |  |  |  | |  | |
|  | M1010-1 | GCK | 7 | - | Exon2-3 |  |  |  |  |  | |  | |

**Figure S1–Workflow of experimental procedure before sequencing.**

Sonicated fragmentation

LM-PCR to check library prep, then hybridize with capture probe

DNA hybridized on capture probes

Wash

Background DNA

Target DNA

Elute

LM-PCR amplification

qPCR and 2100: QC of capture success

Sequencing

**Figure S2–Bioinformatics analysis pipeline.**

1. polluted by adapter
2. containing more than 10% nucleotides out of read length
3. having an average quality of less than 10
4. having 50% bases with a quality value less than 5

Raw reads

49162530bp cleanreads

Mapped to HG19 using BWA

46378487bp unique reads

SOAPsnp software 2.0

In-housepipeline

SAMtools V1.4

CNV

2067 Indel

13808 SNP

Annotated for gene, function, associated disease, and allele frequency in databases

4 in CDS, 1 in splice

70 missense, 18 splice site, 1 nonsense

1. Allele frequency<0.01

2. Gene filtration (six MODY genes)

3. Literature and database searching

4. Functional prediction

Further evaluation

**Figure S3 –Detection of 7 large deletions or duplications in Danish patients with known monogenic diabetes. YH4 is shown as control.**


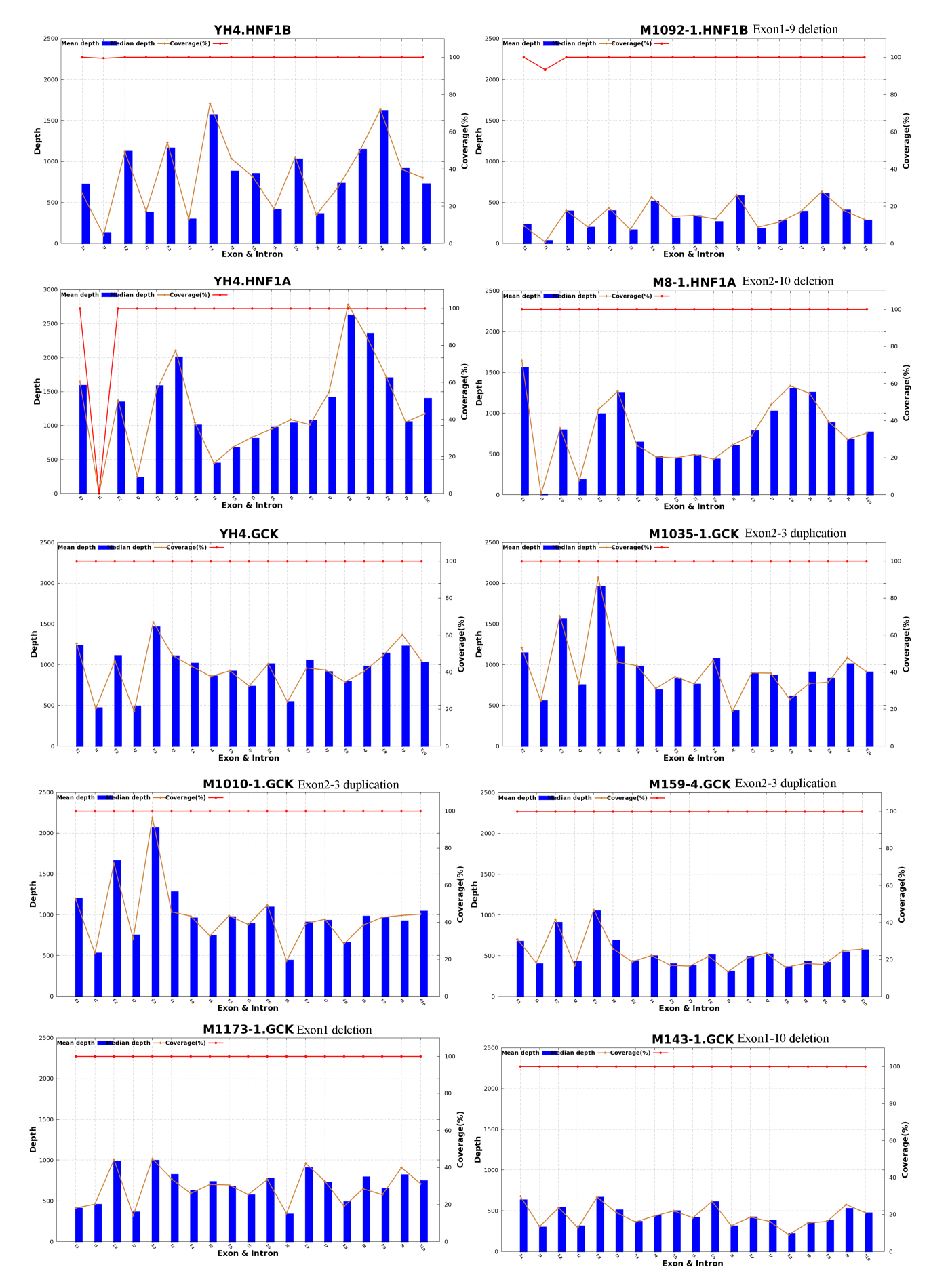


**References:**

1. Gloyn AL: **Glucokinase (GCK) mutations in hyper- and hypoglycemia: maturity-onset diabetes of the young, permanent neonatal diabetes, and hyperinsulinemia of infancy.***Hum Mutat* 2003, **22**(5):353-362.

2. Yamada S, Tomura H, Nishigori H, Sho K, Mabe H, Iwatani N, Takumi T, Kito Y, Moriya N, Muroya K *et al*: **Identification of mutations in the hepatocyte nuclear factor-1alpha gene in Japanese subjects with early-onset NIDDM and functional analysis of the mutant proteins.***Diabetes* 1999, **48**(3):645-648.

3. Jatuporn Sujjitjoon PJWB: **Molecular genetics of monogenetic beta-cell diabetes**. *Thai Journal of Genetics*  2008, **1**(2):93-108.

4. Yamagata K, Oda N, Kaisaki PJ, Menzel S, Furuta H, Vaxillaire M, Southam L, Cox RD, Lathrop GM, Boriraj VV *et al*: **Mutations in the hepatocyte nuclear factor-1alpha gene in maturity-onset diabetes of the young (MODY3)**. *Nature* 1996, **384**(6608):455-458.

5. Chevre JC, Hani EH, Boutin P, Vaxillaire M, Blanche H, Vionnet N, Pardini VC, Timsit J, Larger E, Charpentier G *et al*: **Mutation screening in 18 Caucasian families suggest the existence of other MODY genes.***Diabetologia* 1998, **41**(9):1017-1023.

6. Vilsboll T, Knop FK, Krarup T, Johansen A, Madsbad S, Larsen S, Hansen T, Pedersen O, Holst JJ: **The pathophysiology of diabetes involves a defective amplification of the late-phase insulin response to glucose by glucose-dependent insulinotropic polypeptide-regardless of etiology and phenotype.***J Clin Endocrinol Metab* 2003, **88**(10):4897-4903.

7. Hansen T, Eiberg H, Rouard M, Vaxillaire M, Moller AM, Rasmussen SK, Fridberg M, Urhammer SA, Holst JJ, Almind K *et al*: **Novel MODY3 mutations in the hepatocyte nuclear factor-1alpha gene: evidence for a hyperexcitability of pancreatic beta-cells to intravenous secretagogues in a glucose-tolerant carrier of a P447L mutation.***Diabetes* 1997, **46**(4):726-730.

8. Moller AM, Dalgaard LT, Ambye L, Hansen L, Schmitz O, Hansen T, Pedersen O:**A novel Phe75fsdelT mutation in the hepatocyte nuclear factor-4alpha gene in a Danish pedigree with maturity-onset diabetes of the young.***J Clin Endocrinol Metab* 1999, **84**(1):367-369.

9. Tonooka N, Tomura H, Takahashi Y, Onigata K, Kikuchi N, Horikawa Y, Mori M, Takeda J: **High frequency of mutations in the HNF-1alpha gene in non-obese patients with diabetes of youth in Japanese and identification of a case of digenic inheritance.***Diabetologia* 2002, **45**(12):1709-1712.

10. Galan M, Vincent O, Roncero I, Azriel S, Boix-Pallares P, Delgado-Alvarez E, Diaz-Cadorniga F, Blazquez E, Navas MA: **Effects of novel maturity-onset diabetes of the young (MODY)-associated mutations on glucokinase activity and protein stability.***Biochem J* 2006, **393**(Pt 1):389-396.

11. Shoemaker AH, Zienkiewicz J, Moore DJ: **Clinical assessment of HNF1A and GCK variants and identification of a novel mutation causing MODY2.***Diabetes Res Clin Pract* 2012, **96**(2):e36-e39.

12. Mantovani V, Salardi S, Cerreta V, Bastia D, Cenci M, Ragni L, Zucchini S, Parente R, Cicognani A: **Identification of eight novel glucokinase mutations in Italian children with maturity-onset diabetes of the young.***Hum Mutat* 2003, **22**(4):338.

13. Gidh-Jain M, Takeda J, Xu LZ, Lange AJ, Vionnet N, Stoffel M, Froguel P, Velho G, Sun F, Cohen D *et al*: **Glucokinase mutations associated with non-insulin-dependent (type 2) diabetes mellitus have decreased enzymatic activity: implications for structure/function relationships.***Proc Natl Acad Sci U S A* 1993, **90**(5):1932-1936.

14. Barrio R, Bellanne-Chantelot C, Moreno JC, Morel V, Calle H, Alonso M, Mustieles C: **Nine novel mutations in maturity-onset diabetes of the young (MODY) candidate genes in 22 Spanish families.***J Clin Endocrinol Metab* 2002, **87**(6):2532-2539.

15. Christesen HB, Jacobsen BB, Odili S, Buettger C, Cuesta-Munoz A, Hansen T, Brusgaard K, Massa O, Magnuson MA, Shiota C *et al*: **The second activating glucokinase mutation (A456V): implications for glucose homeostasis and diabetes therapy.***Diabetes* 2002, **51**(4):1240-1246.

16. Johansen A, Ek J, Mortensen HB, Pedersen O, Hansen T: **Half of clinically defined maturity-onset diabetes of the young patients in Denmark do not have mutations in HNF4A, GCK, and TCF1.***J Clin Endocrinol Metab* 2005, **90**(8):4607-4614.

17. Moller AM, Dalgaard LT, Pociot F, Nerup J, Hansen T, Pedersen O: **Mutations in the hepatocyte nuclear factor-1alpha gene in Caucasian families originally classified as having Type I diabetes.***Diabetologia* 1998, **41**(12):1528-1531.

18. Ryffel GU: **Mutations in the human genes encoding the transcription factors of the hepatocyte nuclear factor (HNF)1 and HNF4 families: functional and pathological consequences.***J Mol Endocrinol* 2001, **27**(1):11-29.

19. Radha V, Ek J, Anuradha S, Hansen T, Pedersen O, Mohan V: **Identification of novel variants in the hepatocyte nuclear factor-1alpha gene in South Indian patients with maturity onset diabetes of young.***J Clin Endocrinol Metab* 2009, **94**(6):1959-1965.

20. Ng MC, Cockburn BN, Lindner TH, Yeung VT, Chow CC, So WY, Li JK, Lo YM, Lee ZS, Cockram CS *et al*: **Molecular genetics of diabetes mellitus in Chinese subjects: identification of mutations in glucokinase and hepatocyte nuclear factor-1alpha genes in patients with early-onset type 2 diabetes mellitus/MODY.***Diabet Med* 1999, **16**(11):956-963.

21. Chi YI, Frantz JD, Oh BC, Hansen L, Dhe-Paganon S, Shoelson SE: **Diabetes mutations delineate an atypical POU domain in HNF-1alpha.***Mol Cell* 2002, **10**(5):1129-1137.

22. Bjorkhaug L, Sagen JV, Thorsby P, Sovik O, Molven A, Njolstad PR: **Hepatocyte nuclear factor-1 alpha gene mutations and diabetes in Norway.***J Clin Endocrinol Metab* 2003, **88**(2):920-931.

23. Vaxillaire M, Abderrahmani A, Boutin P, Bailleul B, Froguel P, Yaniv M, Pontoglio M: **Anatomy of a homeoprotein revealed by the analysis of human MODY3 mutations.***J Biol Chem* 1999, **274**(50):35639-35646.

24. Flanagan SE, Kapoor RR, Mali G, Cody D, Murphy N, Schwahn B, Siahanidou T, Banerjee I, Akcay T, Rubio-Cabezas O *et al*: **Diazoxide-responsive hyperinsulinemic hypoglycemia caused by HNF4A gene mutations.***Eur J Endocrinol* 2010, **162**(5):987-992.

25. Pruhova S, Ek J, Lebl J, Sumnik Z, Saudek F, Andel M, Pedersen O, Hansen T: **Genetic epidemiology of MODY in the Czech republic: new mutations in the MODY genes HNF-4alpha, GCK and HNF-1alpha.***Diabetologia* 2003, **46**(2):291-295.

26. Yokoyama A, Katsura S, Ito R, Hashiba W, Sekine H, Fujiki R, Kato S: **Multiple post-translational modifications in hepatocyte nuclear factor 4alpha.***Biochem Biophys Res Commun* 2011, **410**(4):749-753.

27. Bellanne-Chantelot C, Clauin S, Chauveau D, Collin P, Daumont M, Douillard C, Dubois-Laforgue D, Dusselier L, Gautier JF, Jadoul M *et al*: **Large genomic rearrangements in the hepatocyte nuclear factor-1beta (TCF2) gene are the most frequent cause of maturity-onset diabetes of the young type 5.***Diabetes* 2005, **54**(11):3126-3132.

28. Boesgaard TW, Pruhova S, Andersson EA, Cinek O, Obermannova B, Lauenborg J, Damm P, Bergholdt R, Pociot F, Pisinger C *et al*: **Further evidence that mutations in INS can be a rare cause of Maturity-Onset Diabetes of the Young (MODY).***BMC Med Genet* 2010, **11**:42.

29. Fredheim S, Svensson J, Porksen S, Hansen L, Hansen T, Pedersen OB, Mortensen HB, Barbetti F, Nielsen LB: **Intrafamilial Variability of Early-Onset Diabetes due to an INS Mutation.***Case Rep Genet* 2011, **2011**:258978.

30. Gloyn AL, Pearson ER, Antcliff JF, Proks P, Bruining GJ, Slingerland AS, Howard N, Srinivasan S, Silva JM, Molnes J *et al*: **Activating mutations in the gene encoding the ATP-sensitive potassium-channel subunit Kir6.2 and permanent neonatal diabetes.***N Engl J Med* 2004, **350**(18):1838-1849.

31. Bellanne-Chantelot C, Saint-Martin C, Ribeiro MJ, Vaury C, Verkarre V, Arnoux JB, Valayannopoulos V, Gobrecht S, Sempoux C, Rahier J *et al*: **ABCC8 and KCNJ11 molecular spectrum of 109 patients with diazoxide-unresponsive congenital hyperinsulinism.***J Med Genet* 2010, **47**(11):752-759.

32. Toaima D, Nake A, Wendenburg J, Praedicow K, Rohayem J, Engel K, Galler A, Gahr M, Lee-Kirsch MA: **Identification of novel GCK and HNF1A/TCF1 mutations and polymorphisms in German families with maturity-onset diabetes of the young (MODY).***Hum Mutat* 2005, **25**(5):503-504.

33. Barbetti F, Cobo-Vuilleumier N, Dionisi-Vici C, Toni S, Ciampalini P, Massa O, Rodriguez-Bada P, Colombo C, Lenzi L, Garcia-Gimeno MA *et al*: **Opposite clinical phenotypes of glucokinase disease: Description of a novel activating mutation and contiguous inactivating mutations in human glucokinase (GCK) gene.***Mol Endocrinol* 2009, **23**(12):1983-1989.

34. Ellard S: **Hepatocyte nuclear factor 1 alpha (HNF-1 alpha) mutations in maturity-onset diabetes of the young.***Hum Mutat* 2000, **16**(5):377-385.

35. Labate C, Galbo R, Mammì C, C L: **GCK gene mutational analysis and MODY2 monogenic diabetes**. *THE CHILD a journal of pediatrics* 2012, **1**(2).

36. Lehto M, Wipemo C, Ivarsson SA, Lindgren C, Lipsanen-Nyman M, Weng J, Wibell L, Widen E, Tuomi T, Groop L: **High frequency of mutations in MODY and mitochondrial genes in Scandinavian patients with familial early-onset diabetes.***Diabetologia* 1999, **42**(9):1131-1137.

37. Frayling TM, Evans JC, Bulman MP, Pearson E, Allen L, Owen K, Bingham C, Hannemann M, Shepherd M, Ellard S *et al*: **beta-cell genes and diabetes: molecular and clinical characterization of mutations in transcription factors.***Diabetes* 2001, **50 Suppl 1**:S94-S100.

38. Ellard S, Thomas K, Edghill EL, Owens M, Ambye L, Cropper J, Little J, Strachan M, Stride A, Ersoy B *et al*: **Partial and whole gene deletion mutations of the GCK and HNF1A genes in maturity-onset diabetes of the young.***Diabetologia* 2007, **50**(11):2313-2317.
